# Altered Mental Status in Emergency Department Patient with Cerebral Septic Emboli from Infective Endocarditis: Case Report

**DOI:** 10.5811/cpcem.62214

**Published:** 2026-08-06

**Authors:** Andrew G. Theophanous, Rebecca G. Theophanous

**Affiliations:** * University of Toledo, College of Medicine, Toledo, Ohio; † Duke University Health System, Department of Emergency Medicine, Durham, North Carolina; ‡ Durham Veterans Affairs Healthcare System, Durham, North Carolina

**Keywords:** infective endocarditis, altered mental status, brain abscess, septic emboli, case report

## Abstract

**Introduction:**

Infective endocarditis (IE) is associated with high mortality (30%). Patients with structural cardiac disease or implanted hardware have higher risk for IE (23–47%). Diagnosis per the 2023 Duke–International Society for Cardiovascular Infectious Diseases criteria is by pathological confirmation or the major/minor criteria. Major criteria include ≥ 2 positive blood culture sets, echocardiography or computed tomography vegetation visualization, and surgical visualization. Septic emboli symptoms (which complicate 25% of IE cases) include neurological deficits or shortness of breath. Early intravenous antimicrobial therapy within one hour for patients who meet sepsis criteria is recommended per Infectious Diseases Society of America guidelines.

**Case report:**

A middle-aged male with recent IE and aortic valve prosthesis presented to the emergency department with altered mental status and hypoglycemia. He had right basilar lung rales but no heart murmur, leg swelling, or jugular venous distension. He met sepsis criteria with leukocytosis and hypothermia. Computed tomography head was performed due to his altered mental status and revealed a right parietal-occipital hypodense lesion concerning for an abscess with edema and mass effect. Given his history of IE and ill appearance, three blood culture sets were drawn and intravenous antibiotics initiated. The patient was admitted to the hospital with magnetic resonance imaging confirming brain abscess; and neurosurgery performed a craniotomy with brain abscess evacuation. Intravenous antibiotics were continued for four weeks for septic brain emboli from recent IE.

**Conclusion:**

Clinicians should keep a broad differential for altered mental status patients. Sepsis patients should have antimicrobials initiated within one hour. Expedited diagnosis with three sets of blood cultures, echocardiography, and surgical consult should be completed in patients with suspected infective endocarditis for improved patient outcomes. Finally, clinicians should evaluate for septic emboli symptoms such as neurological deficits or respiratory symptoms.

## INTRODUCTION

Infective endocarditis (IE) is associated with a high mortality rate (30%), with over one million cases diagnosed in 2019 and an annual incidence of 13.8 cases per 100,000 person years.^1^,^2^ Infective endocarditis involves infection on an endocardial surface of the heart such as the cardiac valves (native or prosthetic), mural endocardium, septal defect, or indwelling cardiac device. It can be linked to underlying structural cardiac disease such as rheumatic or congenital heart disease. The most common pathogens are *Staphylococcus*, *Streptococcus*, and *Enterococcus* (about 80% of IE cases).^1^ Positive blood culture rate ranged from 45–79% in a comprehensive review. Noncardiac risk factors include older age, male sex, central catheter, intravenous (IV) drug use, immunosuppression, recent dental or surgical procedure, recent hospitalization, and hemodialysis.^1^

Diagnosis involves clinical findings, microbiological results, and imaging.^2^ Clinical examination can reveal fever (77% of cases as per the European Society of Cardiology EurObservational Research Programme European Endocarditis registry), new heart murmur (64%), congestive heart failure (27%), petechiae, Janeway lesions (painless macules on the palms/soles), Osler nodes (painful red lesions on the fingertips), Roth spots (small red-colored retinal hemorrhage with pale centers), and splenomegaly.^1^–^3^ Of patients with IE, 25% develop septic emboli, which can travel to the lungs (presenting with shortness of breath, cough, acute heart failure, etc) and brain (presenting with neurological deficits or altered mental status).^1^–^3^

We describe an emergency department (ED) patient with altered mental status and hypoglycemia found to have septic emboli and brain abscess from IE.

## CASE REPORT

A 65-year-old male with hypertension, emphysema, diabetes mellitus type 2, recent right foot gangrene, dementia, prior stroke, and methicillin sensitive *Staphylococcus aureus* (MSSA) bacteremia with endocarditis status post bioprosthetic aortic valve replacement one month prior presented to the ED from a skilled nursing facility with altered mental status. Per facility staff, he had missed breakfast and was found lying on the floor nonresponsive. He had not had a recent change in his insulin or other medications. He received intramuscular glucagon followed by 25 g IV dextrose by the paramedics for a low blood sugar of 52 mg/dL (reference range, 70–100 mg/dL), after which he became responsive and coughed up secretions.^3.5^ Repeat blood glucose was 83 mg/dL. On ED arrival, his vital signs were as follows: blood pressure, 130/76 mm Hg; heart rate, 88 beats per minute; respiratory rate, 18 breaths per minute; oxygen saturation, 100% on room air; and he was hypothermic at 34.6 °C.

On physical examination, his recent sternotomy site was clean without purulent drainage or erythema. The examination revealed right lower lung lobe rales but no heart murmur. There was no abdominal tenderness, leg edema, or jugular venous distension. His chronic wound on the right foot was wrapped in clean dressings without increased drainage or foul odor. He did not have focal neurological deficits. He was awake and oriented to self, place, and date. Laboratory tests revealed the following: leukocytosis, 12.9x10^9/L (reference range, 4,500–11,000/mm^3); chronic anemia with hemoglobin 8.9 g/dL (13.5–17.5 g/dL [males]); hematocrit 27.9% (41–43%); and no electrolyte abnormalities or renal failure (creatinine 0.7 mg/dL [97–137 mL/min]). Chest radiography showed aspiration pneumonitis. His repeat blood glucose was low in the ED (10 mg/dL), and he was started on a dextrose 10% infusion.

*CPC-EM Capsule*

What do we already know about this clinical entity?

*Infective endocarditis (IE) is associated with high mortality (30%).*

What makes this presentation of disease reportable?

*A 65-yr-old patient who presented with altered mental status was found to have septic cerebral emboli resulting from IE.*

What is the major learning point?

*Clinicians should keep a broad differential for altered mental status patients. Sepsis patients should have antimicrobials initiated within one hour.*

How might this improve emergency medicine practice?

*Expedited diagnosis with three sets of blood cultures, echocardiography, and surgical consult should be completed in patients with suspected IE.*

**Image 1:**
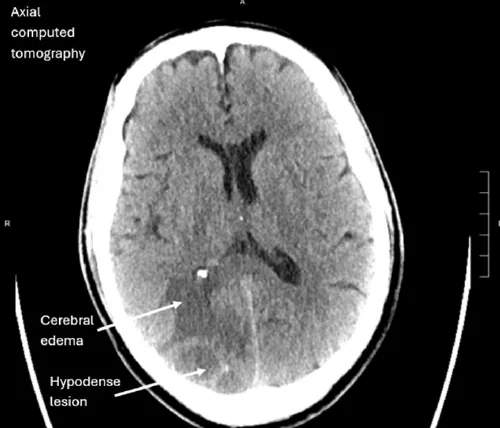
Axial computed tomography head image showing a 2.5 centimeter hypodense lesion in the right parietooccipital lobe with increased hypodensity in the right perirolandic region, concerning for cerebritis and possible intracranial abscess (white arrows).

Head computed tomography (CT), performed because of the patient’s altered mental status, revealed a right parietooccipital 2.5 × 2.0-cm centrally hypoechoic lesion suggestive of an abscess with surrounding edema (Image 1). Because of his history of IE, ill appearance, leukocytosis, and hypothermia, three blood culture specimens were obtained. Empiric therapy IV cefepime and vancomycin was initiated for bacteremia and sepsis.

**Image 2:**
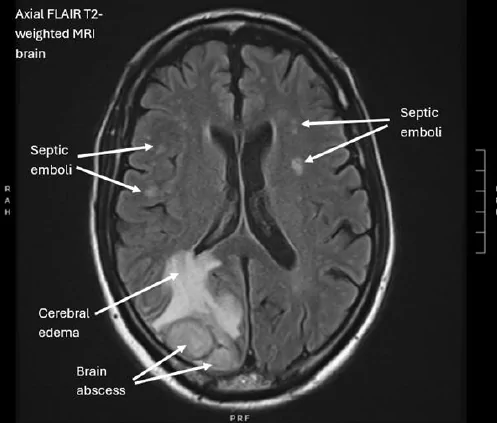
Axial fluid attenuated inversion recovery brain magnetic resonance image showing increased T2-weighted signal in the posterior right frontal lobe and right parietal-occipital region with mild to moderate localized edema and mild mass effect but no midline shift, suspicious for multifocal brain abscess (white arrows).

He was admitted to the hospital for altered mental status and hypoglycemia. Brain magnetic resonance imaging with contrast confirmed multifocal septic emboli complicated by cerebritis and findings suspicious for developing multifocal abscess formation (Image 2).

The most conspicuous lesions were in the posterior right frontal lobe and right parietooccipital region with mild-to-moderate localized edema and mass effect but no midline shift. A burr hole craniotomy was performed that evening by the neurosurgery service using stereotactic neuronavigation (Brainlab) and volumetric evacuation of the brain abscess, which subsequently grew MSSA. He was transferred to the neurological intensive care unit for serial neurologic assessments and was initiated on levetiracetam 500 mg twice daily for seizure prophylaxis. Transthoracic echocardiogram (TTE) with bubble study demonstrated a normal ejection fraction > 55%, moderate mitral regurgitation, well-seated bioprosthetic aortic valve with paravalvular thickening but no vegetative mass, and a trivial pericardial effusion.

Chest CT angiogram with contrast did not show cardiac abnormalities, aortic root abscess, or pulmonary embolism. By cardiothoracic surgery recommendations, a transesophageal echocardiogram was not performed because there was no root abscess at the time of valve implant and the TTE had clear views of the valves; thus, they did not suspect recurrent IE. While blood cultures did not grow any bacteria, the brain abscess did grow MSSA from intraoperative cultures. By infectious diseases consult recommendations, the patient was continued on IV cefazolin for one week and discharged to a skilled nursing facility with IV nafcillin via a tunneled catheter for central nervous system septic emboli to complete a four-week antibiotic course.

## DISCUSSION

Emergency physicians should keep a broad differential diagnosis for patients with altered mental status. They should perform a thorough clinical examination including full neurologic testing for focal deficits, which can localize to specific affected areas of the brain or spinal cord. The 1-year, 5-year, and 10-year mortality rates of patients with sepsis and IE are high (22.7%, 37.5%, and 48.5%, respectively).^4^ Clinicians should have heightened suspicion for sepsis in undifferentiated patients, since early antibiotic treatment within one hour significantly reduces morbidity and mortality.^5^ Patients who present with hypoglycemia, hypothermia, confusion, or chills can be exhibiting signs of systemic infection or sepsis.^5^

In patients with IE, physicians should look for clinical signs of symptomatic embolic complications such as neurological changes with central nervous system)involvement or shortness of breath or hypoxia with pulmonary involvement (25% of IE cases had emboli in a comprehensive review).^1^,^2^ Because the patient had altered mental status, neuroimaging was performed to evaluate for potential causes such as hemorrhage, mass, infarction, or infection. The most common emboli from IE are cerebral (occurring in 25% of cases and diagnosed in this patient), pulmonary (6.4%), splenic (5.7%), renal (2.5%), hepatic (0.5%), and peripheral (3%).^1^,^6^,^7^ Rates are higher in patients with a pacemaker (23%) or prosthetic valve (44–47%).^6^,^7^ Although the patient did not report any visual changes, headache, or vomiting, the brain lesions were localized to the parietooccipital region. A more comprehensive visual examination or formal ambulatory gait testing may have potentially detected the occipital lobe involvement of the abscess.

Patients can also develop cardiac complications from IE including heart block (3–8%) or a paravalvular abscess (12%). Right-sided IE is more likely to cause pulmonary septic emboli, whereas left-sided IE causes brain emboli based on blood circulatory pathways. Pathologic diagnosis is the gold standard. Patients with underlying structural heart disease or artificial valves are susceptible to culture negative bacteria of the HACEK group (*Haemophilus, Aggregatibacter, Cardiobacterium, Eikenella corrodens, and Kingella*). Immunosuppressed individuals may have fungal infections.^1^,^3^ Transesophageal echocardiography is the gold standard radiographic modality for IE diagnosis, although noninvasive TTE is typically performed first to detect vegetations, as was done in this patient case (specificity > 90% and sensitivity 75%). Cardiac CT can assess paravalvular and periprosthetic complications.^1^,^3^,^6^,^7^

The updated 2023 Duke–International Society for Cardiovascular Infectious Diseases criteria have high sensitivity (84%) for IE diagnosis.^3^ Definite IE is defined by pathologic confirmation or clinically as meeting (1) two major criteria (microbiology, diagnostic imaging, or surgical); (2) one major criterion and three minor criteria; or (3) five minor criteria (Table 1).^3^

Intravenous antibiotics should be initiated after blood cultures are drawn in high-risk patients with high clinical suspicion for improved clinical outcomes, with a treatment duration of four to six weeks in confirmed cases.^1^,^2^ Per sepsis criteria, antibiotics should be started within 60 minutes of ED presentation.^5^ With early and adequate treatment, survival rates are good (85–90% at 1 year and 70–80% at 5 years). Infective endocarditis can recur at rates between 2–9%. Treatment for relapse involves another four to six weeks of IV antibiotics per the American Heart Association IE guidelines, with consideration of cardiac surgery if source control is needed.^2^ Table 2 highlights the main educational points regarding ED management of sepsis, IE, and altered mental status related to this patient’s care.^5^,^8^,^9^

## CONCLUSION

Our patient was treated for cerebral septic emboli and cerebritis as a complication of recent infective endocarditis with four weeks of intravenous antibiotics. Clinicians should draw three blood culture sets in cases of suspected IE, obtain transthoracic echocardiogram or transesophageal echocardiogram for diagnostic imaging confirmation, and initiate antimicrobials within one hour in patients meeting sepsis criteria for optimal patient outcomes. Also, clinicians should evaluate for septic emboli symptoms such as neurological deficits with central nervous system involvement and respiratory symptoms with lung involvement in IE patients.

## Figures and Tables

**Table 1:** Summary of the 2023 Duke–International Society for Cardiovascular Infectious Diseases criteria for diagnosis of infective endocarditis.

Clinical criteria	Microbiology	Diagnostic imaging	Surgical/pathological
Major criteria	2 or 3 positive blood culture sets with the same organism	Vegetation or mass visualized on echocardiography or cardiac computed tomography	Active endocarditis identified by vegetation sample
	Positive polymerase chain reaction test for *Coxiella burnetii*, *Bartonella* species, or *Tropheryma whipplei* from blood	Abnormal metabolic activity on positron emission computed tomography with 18F-fluorodeoxyglucose	Direct visualization of IE from surgical specimen
Minor criteria	New cardiac murmur, fever, vascular phenomena (arterial emboli, septic pulmonary infarcts, cerebral or splenic abscess, mycotic aneurysm, intracranial hemorrhage, conjunctival hemorrhages, Janeway lesions, purulent purpura), predisposing factors (previous history of IE, prosthetic valve or valve repair, congenital heart disease, moderate-severe valve regurgitation or stenosis, endovascular intracardiac implantable device, hypertrophic obstructive cardiomyopathy, injection drug use)		

*IE*, infective endocarditis.

**Table 2:** Main educational points for emergency department management of sepsis and septic shock, infective endocarditis, and altered mental status in adult patients.

Causes of illness	Laboratory diagnostics	Imaging diagnostics	Treatment
Sepsis/septic shock: Urinary tract/renal infection, pneumonia, osteomyelitis, bloodstream or central nervous system infection	Send two sets of blood cultures within 1 hour Urine test Consider respiratory specimen or viral swab	Computed tomography or radiographs	Initiate broad spectrum IV antibiotics within 1 hour of ED arrival IV fluids (30cc/kg unless contraindicated) Vasopressors to maintain mean arterial pressure (> 65 mm Hg)
Infective endocarditis: native valve with deformity, implanted cardiac hardware. Septic emboli to brain, lung, renal, spleen, liver, or peripheral	Send three sets of blood cultures	Transthoracic echocardiogram (TTE); then transesophageal echocardiogram if high clinical suspicion or high-risk TTE features to detect complications XR/CT/MRI of head, chest or abdomen, or extremities based on clinical suspicion for septic emboli	Source control with surgery or removal of hardware Total duration of 4–6 weeks IV antibiotics
Altered mental status: from CNS infection, toxidrome, medications, or neurological process	Add toxicology screens Review medication adverse effects or polypharmacy Consider lumbar puncture after head imaging Evaluate for focal neurological deficits in cases of suspected stroke or intracranial hemorrhage	CT or MRI of the brain	IV antibiotics and fluids in cases of infection Adjust medications if needed Treat toxidrome as indicated by poison control center Thrombolytics or interventional treatment for stroke Hemorrhage reversal or neurosurgical intervention

*CT*, computed tomography; *CNS*, central nervous system; *IV*, intravenous; MRI, magnetic resonance imaging; *XR*, radiograph; *ED*, Emergency Department; *mmHg*, millimeters of mercury; *cc/kg*, cubic centimeters per kilogram.
